# Hippocampal volume and cognitive performance in children with congenital heart disease

**DOI:** 10.1038/s41390-022-02457-2

**Published:** 2023-01-07

**Authors:** Nadja Naef, Amélie Ciernik, Beatrice Latal, Rabia Liamlahi, For the Children’s Heart and Development Research Group

**Affiliations:** 1 Child Development Center, University Children’s Hospital Zurich, Zurich, Switzerland; 2 Children’s Research Center, University Children’s Hospital Zurich, Zurich, Switzerland

## Abstract

**Background:**

Congenital heart disease (CHD) is associated with an increased risk of brain abnormalities. Studies indicate a particular vulnerability of the hippocampus to hypoxia and inflammation. Yet, information regarding the hippocampus and its relation to cognitive function in school-age children with CHD remains scarce.

**Methods:**

Children who underwent cardiopulmonary bypass surgery for CHD (*N* = 17) and healthy controls (*N* = 14) at 10 years of age underwent neurodevelopmental assessment and cerebral magnetic resonance imaging to measure IQ, working memory performance and hippocampal volume.

**Results:**

IQ was significantly lower in children with CHD compared to controls (98 vs 112, *P* = 0.02). Children with CHD showed worse working memory performance with significantly lower scores in the letter-number sequencing test (*P* = 0.02). After adjusting for total brain volume, hippocampal volume was smaller in children with CHD compared to controls (*P* < 0.01). Smaller hippocampal volume was associated with lower IQ (*P* = 0.04), and digit span scaled score (*P* = 0.03), but not with other working memory tests (*P* > 0.1).

**Conclusion:**

This study suggests that the hippocampus may be particularly susceptible in children with CHD thereby contributing to cognitive impairments. Further research is necessary to understand the contribution of the hippocampus to cognitive impairments in children with CHD.

**Impact:**

- IQ is significantly lower in school-age children with congenital heart disease compared to controls.
- Working memory performance seems to be worse in children with congenital heart disease.
- Smaller hippocampal volume is associated with lower IQ and seems to be associated with lower working memory performance.
- The study adds knowledge on the etiology of cognitive impairments in school-age children with congenital heart disease.

## Introduction

Children with congenital heart disease who undergo open-heart surgery are at increased risk for mild to moderate impairments of neurodevelopmental outcome.^1^ Cognitive outcome and in particular working memory performance have been shown to be affected in children with CHD.^1^ As working memory abilities are crucial for academic achievement they should be specifically assessed.^2^

The etiology of the observed cognitive and working memory impairments in children with CHD is not fully understood. There is evidence for a particular vulnerability of the hippocampus in the CHD population likely due to neuroinflammation and hypoxia resulting from critical illness in the neonatal period.^3–7^ In this small cohort study, we aimed to investigate the relationship between working memory impairments in children with CHD and hippocampal alterations.

## Methods

### Study participants

In this study, 26 children with CHD and 17 controls (typically developing children, cross-sectional recruitment) between 9 and 11 years of age were included. The study was approved by the ethics committee of the Canton of Zurich, Switzerland, and written informed consent was obtained from the legal guardians. Children with CHD were recruited between July 2016 and July 2019 from the prospective Research and Child Health Outcome (ReachOut) study.^8^ All children underwent cardiopulmonary bypass surgery between 2004 and 2009 and were followed-up until 10 years of age if they had no genetic disorder or dysmorphic syndrome.^8^ Neurodevelopmental assessment was performed in all participants and cerebral MRI was obtained in 34 (CHD *N* = 18).

### Measurements

#### Neurodevelopmental assessment

To estimate the IQ a short form of the Wechsler intelligence scale for Children 4th edition (WISC-IV), German version was applied (subtests applied: working memory, processing speed, mosaic test, letter-number sequencing, digit symbol test, vocabulary and symbol search).^9,10^ Working memory was measured with the test for attentional performance (TAP) working memory,^11^ the digit span (WISC-IV), the letter-number sequencing (WISC-IV) and the Corsi block-tapping test.^12^ The sum of commission and omission errors from the TAP working memory test was used, where higher number of errors indicated worse task performance. The digit span and letter-number sequencing test were expressed as scaled scores. To estimate visual-spatial working memory a forward and backward trial were combined in the Corsi block-tapping test. Socio-economic status (SES) was estimated based on maternal education and paternal profession (range 2 - 12). Medical and demographic data were collected through questionnaires or from patient charts.

### Brain imaging

Three-dimensional anatomic images were obtained using T1 weighted gradient echo pulse sequence with a 3 T GE MR750 scanner. Cortical reconstruction and volumetric segmentation were performed with the FreeSurfer image analysis suite version 5.3.0, which is documented and freely available for download online (http://surfer.nmr.mgh.harvard.edu/). Segmentation of total brain volume (TBV) and hippocampal volume was successful in 31 participants (CHD *N* = 17).

### Statistics

Participant characteristics and outcome measures are presented as mean and standard deviation (SD) or median and interquartile range (IQR). Group differences were calculated with the *t*-test or Mann–Whitney-*U*-Test for numeric and the X2 test for categorical variables. The association of hippocampal volume with cognitive outcome (IQ, working memory) was assessed in the combined group (children with CHD and controls) using linear regression with cognitive outcome as the dependent and hippocampal volume as independent variable, adjusted for age, sex, SES, and group. Two-tailed *P* < 0.05 were considered significant. Analysis was conducted with the R software (R core team, version 4.0.2, URL http://Rproject.org/).

## Results

### Neurocognitive performance

Subject characteristics and neurocognitive outcome are presented in Table 1. Children with CHD showed significantly lower IQ scores compared to controls, after adjusting for age, sex, and SES (*β* = −0.41, SE = 0.2, *P* = 0.02). Scores of working memory performance were lower in all tests in the CHD group and errors on the TAP task were higher compared to controls, but did not reach significance (*P* > 0.1). Only in the letter number sequencing test children with CHD performed significantly worse compared to controls (*P* = 0.02), but not after adjusting for age, sex, and SES (−0.32, SE = 0.2, *P* = 0.12).

**Table 1:** Subject characteristics and neurocognitive performance.

	CHD, *N* = 17	Controls, *N* = 14	*P*	
Sex (male), *N* (%)	12 (70.6)	8 (57.1)	0.69	
Age, Mean (SD)	10.8 (0.37)	10.3 (0.78)	0.03	
SES, Median (IQR)	8.0 (7; 10)	8.5 (7.75; 10)	0.82	
Univentricular CHD, *N* (%)	2 (11.8)			
Cyanotic CHD, *N* (%)	14 (82.4)			
Age 1st bypass surgery, months, Mean (SD)	1.1 (1.2)			
One bypass surgery, *N* (%)	13 (76.5)			
Diagnosis				
d-TGA	8 (47.1)			
Other	9 (52.9)			
Neurocognitive outcome				Cohens‘ d
IQ	98.24 (12.42)	111.86 (8.38)	0.003	1.29
Corsi block tapping test total score	16.44 (3.50)	17.50 (3.48)	0.41	0.31
Digit span scaled score	9.80 (2.60)	10.79 (3.09)	0.36	0.36
Letter number sequencing scaled score	9.73 (3.03)	12.38 (2.66)	0.02	0.96
TAP working memory total error	8.38 (5.76)	7.70 (6.07)	0.78	−0.12

Cohens’ d Effect size: small (0.2), medium (0.5) and large (0.8).

*CHD* Congenital heart disease, *SD* Standard deviation, *SES* Socio economic status, *IQR* Interquartile range, *d-TGA* d-Transposition of the great arteries, *IQ* Intelligence quotient, *TAP* Test of attentional performance.

### Total brain volume and hippocampal volume

**Fig. 1:**
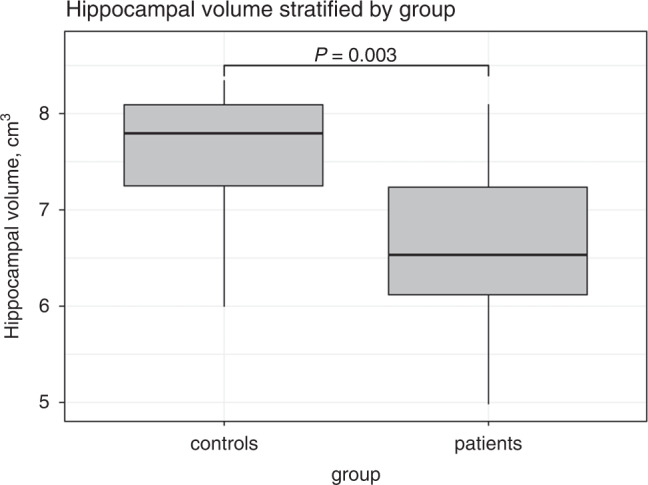
Boxplot of hippocampal volumes for children with CHD and controls plotted separately. The boxplots illustrate hippocampal volume in cm^3 plotted for controls and children with CHD separately. In a linear regression model, controlling for sex, age and SES, hippocampal volume was significantly smaller in children with CHD compared to controls (*P* = 0.003).

TBV was not significantly different between children with CHD and controls (1073 (108) vs 1115 (119) cm^3, *β* = −0.29, SE = 0.2, *P* = 0.16, adjusted for age, sex and SES). Total hippocampal volume was smaller in children with CHD compared to controls (6.68 (0.79) vs 7.56 (0.72), *β* = −0.60, SE = 0.2, *P* = 0.003, adjusted for age, sex and SES), see Fig. 1. Similarly, the right and left hippocampus were smaller in children with CHD compared to controls (both *P* < 0.01). The difference in total hippocampal volume remained significant after adjusting for TBV (*P* = 0.007). Cardiac diagnosis was not associated with hippocampal volume (univentricular, cyanotic: both *P* > 0.1).

### Association of total hippocampal volume with cognitive performance

**Fig. 2:**
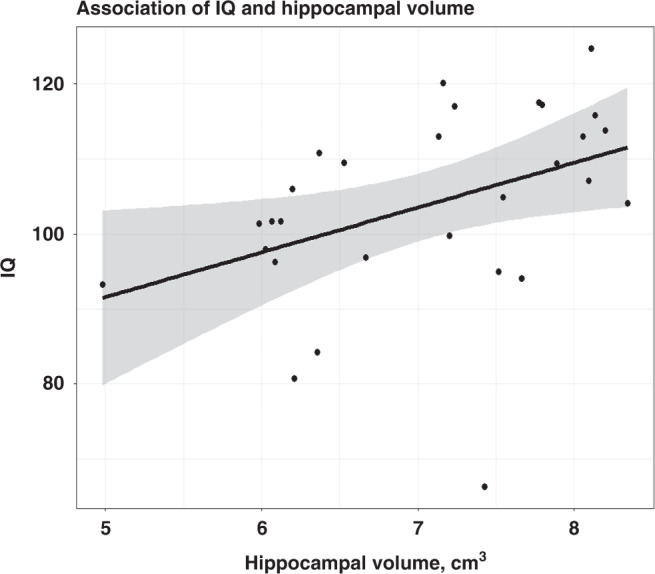
Association of total hippocampal volume and IQ. Figure depicts the association of total hippocampal volume and IQ for the combined group (CHD and controls). The regression line has been fitted using linear regression. Larger hippocampal volume was positively associated with higher IQ (*P* = 0.04), adjusted for age, sex, SES, and group.

In the combined group, smaller total hippocampal volume was significantly associated with lower IQ (*P* = 0.04), but not after adjusting for TBV (*P* = 0.13), see Table 2 and Fig. 2. Adding TBV to the regression model did not contribute to a better model fit (*P* = 0.99). Total brain volume was not associated with lower IQ (*β* = 0.23, SE = 0.16, *P* = 0.180). Smaller hippocampal volume was associated with a lower digit span scaled score (*P* = 0.031), but not with the other working memory tests (*P* > 0.1), see Table 2. We have re-calculated the models in Table 2 including an interaction of group and total hippocampal volume. IQ and digit span were associated with total hippocampal volume (*P* < 0.05). Of note, all interactions were non-significant (*P* > 0.05).

**Table 2:** Total hippocampal volume in relation to cognitive outcome and working memory performance.

	Total hippocampal volume					
	R^2	Estimate	CI	*β*	SE	*P*
IQ	0.477	5.58	0.28; 10.88	0.38	0.18	0.04
Corsi block	0.187	0.96	−0.88; 2.79	0.23	0.22	0.29
Digit span	0.328	1.46	0.15; 2.77	0.46	0.20	0.03
Letter-number sequencing	0.130	0.80	−0.90; 2.49	0.22	0.23	0.34
TAP working memory total error	0.521	−1.64	−4.27; 0.99	−0.24	0.19	0.20

Linear regression analyses (dependent variable: functional outcome, independent variable: hippocampal volume; adjusted for sex, age and SES).

## Discussion

In this small study, we found evidence of smaller hippocampal volumes in school-aged children who underwent cardiopulmonary bypass surgery for CHD. Hippocampal volume reduction was associated with worse IQ and digit span score in both children with CHD and controls. Our findings provide further evidence for the particular vulnerability of the hippocampal structure and its contribution to cognitive impairments in the CHD population.

In our CHD cohort, we found smaller hippocampal volumes, after controlling for differences in total brain volume. Recent research suggests a particular vulnerability of the hippocampus in the CHD population with hippocampal volume reductions in fetuses and newborns with CHD.^3–6^ The hippocampus is thought to be selectively vulnerable to neuroinflammation and hypoxia conditions which are found in children with critical illness in the neonatal period such as CHD or extremely premature birth and severe respiratory failure.^7^ Another study in children with transposition of the great arteries (TGA) found abnormal hippocampal volume in only 40% of children, despite neonatal hypoxia inherent to TGA.^13^ Thus, hypoxia and neuroinflammation may not fully explain hippocampal alterations in the CHD population. In healthy fetuses with a family history of CHD, hippocampal volumes were reduced similar to fetuses with CHD indicating that genetic and environmental factors may independently contribute to hippocampal alterations.^3^ Another study found higher maternal stress associated to smaller hippocampal volume in mothers carrying fetuses with CHD.^5^ Thus, hippocampal alterations are likely a result of a synergy of various in part modifiable factors.

Similarly, studies in children and young adults with CHD found brain volumetric alterations, indicating long-term hippocampal impairments likely resulting from neonatal critical illness.^6,7,13–15^ We were able to confirm this finding in our more recent cohort suggesting that hippocampal alterations are still apparent in children with CHD despite continued advances in medical care.

Smaller hippocampal volume is associated with lower general cognitive abilities and memory functions, including working memory in CHD patients.^6,13,14^ We could replicate the association of hippocampal volume and IQ in the combined sample of children with CHD and controls. However, when adjusting for TBV, the association became non-significant, indicating that smaller hippocampal volume may serve as a proxy for smaller TBV. We tried to account for this by comparing the two models (with and without adjusting for TBV) and were able to show that the model fit decreased when adjusting for TBV. In addition, TBV was similar between patients and controls and was not associated to IQ in our combined sample of children with CHD and controls. Thus, smaller hippocampal volumes may contribute to IQ irrespective of TBV.

When investigating the association of hippocampal volume and working memory, we only found an association of reduced hippocampal volume with worse performance on the digit span test. Due to our small sample size, we may not have been able to detect associations with the other working memory tests. Possibly, the association of hippocampal volume and functional impairment may vary among individuals, as previously noted by Muñoz-López and colleagues, who found inconsistent association of hippocampal volume reduction with memory impairment in children with TGA.^13^ This variability in function-structure relationship may be due to the limited sensitivity of whole hippocampus segmentation. In one study in CHD adults, smaller left cornu ammonis area 1 (CA1) volume and left CA2/3 volume were associated with worse working memory ability.^14^ They further reported altered hippocampal shape in CHD adults compared to controls.^14^

IQ difference between children with CHD and controls in our study was smaller than anticipated based on previous studies.^1^ Our study design with MR imaging and lengthy cognitive assessment may have resulted in a selection bias towards high functioning children with CHD, as children with school difficulties or extracurricular tutoring may have refused to participate due to lack of time. Further, we recruited two siblings of children with CHD as controls, which may have led to an underestimation of hippocampal differences due to similarities in genetic and environmental factors.^3^ Due to the small sample size, statistical power was limited and may have hindered our ability to detect associations between hippocampal volume and working memory performance. Due to the lack of power, we were not able to investigate potential group differences in the association of hippocampal volume and cognitive outcome. Thus, the results of this study should be interpreted accordingly. Despite these limitations, hippocampal volume was smaller in our recent cohort of CHD children compared to controls and was associated to cognitive functions. The hippocampus seems to be particularly vulnerable in the CHD population. Further research is necessary to understand the contribution of the hippocampus to cognitive function and working memory performance.

## Data Availability

The datasets generated during and/or analysed during the current study are available from the corresponding author on reasonable request.
